# The Potential Coordination of the Heat-Shock Proteins and Antioxidant Enzyme Genes of *Aphidius gifuensis* in Response to Thermal Stress

**DOI:** 10.3389/fphys.2017.00976

**Published:** 2017-11-28

**Authors:** Zhi-Wei Kang, Fang-Hua Liu, Xiang Liu, Wen-Bo Yu, Xiao-Ling Tan, Shi-Ze Zhang, Hong-Gang Tian, Tong-Xian Liu

**Affiliations:** ^1^State Key Laboratory of Crop Stress Biology for the Arid Areas, and Key Laboratory of Northwest Loess Plateau Crop Pest Management of Ministry of Agriculture, Northwest A&F University, Yangling, China; ^2^State Key Laboratory of Integrated Management of Pest and Rodents, Institute of Zoology, Chinese Academy of Sciences, Beijing, China; ^3^Entomology Department, College of Plant Protection, Yunnan Agricultural University, Kunming, China; ^4^State Key Laboratory for Biology of Plant Diseases and Insect Pests, Institute of Plant Protection, Chinese Academy of Agricultural Sciences, Beijing, China

**Keywords:** *Aphidius gifuensis*, heat tolerance, heat-shock protein, antioxidant enzymes, integrated pest control

## Abstract

*Aphidius gifuensis* is one of the most important aphid natural enemies and has been successfully used to control *Myzys persicae* and other aphid species. High temperature in summer is one of the key barriers for the application of *A. gifuensis* in the field and greenhouse. In this work, we investigated the biological performance of *A. gifuensis* and the response of heat-shock proteins and antioxidant enzymes under high temperature. The results showed that *A. gifuensis* could not survive at 40°C and female exhibited a higher survival in 35°C. Furthermore, the short term exposure to high temperature negatively affected the performance of *A. gifuensis* especially parasitism efficiency. Under short-term heating, the expression of *AgifsHSP, Agifl(2)efl, AgifHSP70, AgifHSP70-4* and *AgifHSP90* showed an increased trend, whereas *AgifHSP10* initially increased and then decreased. In 35°C, the expressions of *Agifl(2)efl, AgifHSP70-4* and *AgifHSP90* in female were higher than those in male, whereas the expression of *AgifHSP70* exhibited an opposite trend. Besides the HSPs, we also quantified the expression levels of 11 antioxidant enzyme genes: *AgifPOD, AgifSOD1, AgifSOD2, AgifSOD3, AgifCAT1, AgifCAT2, AgifGST1, AgifGST2, AgifGST3, AgifGST4* and *AgifGST5*. We found that the sex-specific expression of *AgifSOD2, AgifSOD3, AgifPOD, AgifGST1* and *AgifGST3* were highly consistent with sex-specific heat shock survival rates at 35°C. Furthermore, when the temperature was above 30°C, the activities of GST, SOD, CAT and POD were significantly increased; however, there was no significant difference of the CAT activity between the male and female at 35°C. Collectively, all of these results suggested that the protection of thermal damage is coordinated by HSPs and antioxidant enzymes in *A. gifuensis*. Based on the heat tolerance abilities of many aphid natural enemies, we also discussed an integrated application strategy of many aphid enemies in summer.

## Introduction

*Aphidius gifuensis* Ashmead (Hymenoptera: Braconidae) is a common solitary endoparasitoid of many agricultural and horticultural pest aphids including *Myzus persicae* (Sulzer), *Aulacorthum solani* (Kaltenbach) and *Sitobion avenae* (Fabricius) (Yang et al., 2011; Pan and Liu, 2014). Due to its highly parasitic efficiency, it has already been successfully used to control *M. persicae* on tobacco in China and been considered to be a potential biological-control agent for effective IPM programs in field and greenhouse (Yang et al., 2011).

Under natural conditions, there are several important determinants restricting the efficiency of the application of *A. gifuensis* including high temperature and raining (Liu et al., 2016). Generally, the temperatures under greenhouse and field conditions showed irregular cyclic variation daily, and the midday temperature often exceeds 40°C for a period of time. The brief heat stress could cause a degree of physiological and ecological damage to pests and their natural enemies (Dong et al., 2013; Sentis et al., 2015). When temperature was higher than 35°C, no female progeny were produced in *Cotesia vestalis* (Shi et al., 2013). In *Aphelinus asychis*, both of the adult survival and longevity are decreased significantly when the temperature was above the 37.5°C (Wang et al., 2016). Furthermore, the decreased mummified aphids and female progeny were also detected at this temperature in *A. asychis* (Wang et al., 2016). Therefore, in greenhouse, the high temperature in summer is the key limiting factor in the application of natural enemies.

Numerous studies of insect-thermal stress interaction have revealed that insect have evolved complex protective mechanism to protect themselves against the high temperature. Heat-shock proteins (HSPs) and antioxidant enzymes were the most well-known effectors in this process (Yang et al., 2010; King and MacRae, 2015).

HSPs are well known as stress proteins and molecular chaperones participating in protein folding, localization and degradation to influence essential process such as protein synthesis, cell signaling, transcription, and metabolism (Feder and Hoffmann, 1999; Sørensen et al., 2003; King and MacRae, 2015). On the basis of molecular mass and sequence homology, HSPs have been divided into several families including the small heat-shock proteins (sHSPs, the molecular weights ranging from 12 to 43 kDa), HSP60, HSP70, HSP90, and HSP10 (Feder and Hoffmann, 1999; Shi et al., 2013; Nguyen et al., 2016). sHSPs are well distributed across tissues and thought to be the first line of cell defense by preventing irreversible denaturation of substrate proteins under biotic and abiotic stress conditions (Kim et al., 1998; Feder and Hoffmann, 1999; Sørensen et al., 2003). Compared to other HSPs, sHSPs exhibit a greater variation in sequence, structure, size, and function. HSP70s have be divided into inducible (Hsp70) and cognate forms (Hsc70s), which are involved in protein translation, folding, unfolding, translocation, and degradation (Qiu et al., 2006). HSP90s participate in the folding, maintenance of structural integrity, and the proper regulation of a subset of cytosolic proteins, and account for 1% of the soluble protein in most tissues, even in the absence of stress (Picard, 2002).

As stress proteins, HSPs are involved in protecting proteins under oxidation and hypertonic stress, which were induced by extreme temperatures, UV, xenobiotic exposures and parasitoid infestation (Shi et al., 2013; Baruah et al., 2014; Zhang L. J. et al., 2015; Chen et al., 2016). For example, in *Harmonia axyridis*, sHSPs were thought to play important roles in the cold hardness process (Wang et al., 2017). The gene expression of HSP70 gene family in *Rhopalosiphum padi* and *Melitatea cinxia* was significantly induced under thermal stress (Luo et al., 2015; Li et al., 2017). Besides the well investigated stress responses, recently research progresses have revealed that HSPs are associated with diverse molecular and physiological functions such as oogenesis, embryo development, diapause and signal transduction (Sørensen et al., 2003; King and MacRae, 2015). For example, knock down of HSP83 in *Acyrthosiphon pisum* significantly reduced its fecundity, longevity and the number of viviparous offspring (Will et al., 2017).

Apart from HSPs, antioxidant enzymes in insect including superoxide dismutase (SOD), catalase (CAT), peroxidases (POD) and glutathione-S-transferases (GST) are the other immune system involved in the oxidative damage response (Lopez-Martinez et al., 2008). These antioxidant enzymes can scavenge the thermal stress, UV, xenobiotic exposures and parasitoid infestation induced surplus reactive oxygen species (ROS) (Lopez-Martinez et al., 2008; Yang et al., 2010; López-Martínez and Hahn, 2012; Wang et al., 2012; Zhang S. Z. et al., 2015; Ali et al., 2016). SOD is the most important antioxidant enzymes in the enzyme defense system against ROS. SOD catalyses the disputation of superoxide radicals into oxygen (O_2)_ and hydrogen peroxide (H_2_O_2_); then H_2_O_2_ is converted by both CAT and POD into oxygen and water (H_2_O).

In previous work, the effect of low temperature (supercooling point and freezing point) on the parasitic potential of *A. gifuensis* was studied (Liu et al., 2016). In this work, we evaluated the biological performance of *A. gifuensis* under a short heat stress. Based on our results and previous studies, we could suggest an integrated pest management strategy with complementary diverse natural enemies. In addition, we unraveled the potential contribution of HSPs and antioxidant enzymes to thermal stress in *A. gifuensis*.

## Materials and methods

### Insect species

*Aphidius gifuensis* were originally collected from *S. avenae* in wheat, Yangling, Shaanxi, China in 2013. The laboratory colony was established and maintained on *S. avenae* at 25 ± 1°C with a 16 h light: 8 h dark photoperiod. *S. avenae* was maintained on winter wheat (*Triticum aestivum* L. Var. “Xinong 979”) in an air-conditioned insectary at 23 ± 1°C, a photoperiod of L16:D8, and relative humidity of 60 ± 5%.

### Thermal stress treatment

For thermal treatments (25°C -control, 30°, 35°, and 40°C, based on temperature detection in greenhouse), groups of 80 pupae and new emerged (1-day-old) adults were collected and placed into a cage (size: 2.4 cm in diameter by 8 cm in height) with a water-wet cotton ball so supply moisture. The cages were covered with nylon gauze (40 meshes) to prevent the escape. The stress duration was set at a selected temperature for 1 h. After thermal treatment, all the treated pupae and adults were separated into two groups: one group with at least 15 living parasitoids were flash-frozen in liquid nitrogen and stored at −80°C until RNA exaction; the rest parasitoids in the other groups were paced for 1 h at 25°C and the hatching rate and survival of *A. gifuensis* were recorded. Each treatment was replicated 3 times.

### Performance of *A. gifuensis* exposure to thermal stress

To keep chili pepper leaf discs (3 cm in diameter) fresh, 3 ml water agar (1%) was poured into a Petri dish (3 cm in diameter and 1.5 cm in height). After brief refrigeration (20–30 min), leaf discs were individually placed on top of the agar in each Petri dish. Then, 30 second- to third- instar *M. persicae* reared on chili pepper plant were placed on the leaf disc in each Petri dish to test the parasitic capacity of *A. gifuensis*. Next day, additional 50 nymphs were provided to each pair of the parasitoids daily for 7 days. The parasitized aphid nymphs were kept in the Petri dishes and held in an incubator at 25 ± 2°C, 70 ± 10% RH, and a photoperiod of 14:10 (L:D) h, allowed to develop until the parasitized aphids mummified. Ten days later, the proportion and number of successful parasitized aphids were recorded. To test the influence of thermal stress on the longevity of *A. gifuensis*, 20 female and male were introduced into a 4.5L plastic cage and provided with plant and *M. persicae*. Parasitoid survival was recorded daily.

### Identification of heat-shock proteins (HSPs) and antioxidant enzymes genes in *A. gifuensis*

Based on the functional identification of *A. gifuensis* transcriptome data, we identified the candidate HSPs and antioxidant enzymes. Then the amino acid sequences of these obtained genes were used to re-Blastp in NCBI to verify the identity with *E*-value < 1e-5.

The functional domains and motifs of obtained genes were identified using the programs ScanProsite, Motifscan and SignalP4.0 online (http://www.cbs.dtu.dk/services/SignalP/). The amino acid sequences of these genes were aligned using MAFFT, with FFT-NS-I iterative refinement method with JTT200 scoring matrix, unalignlevel 0.3, “leave gappy regions” set, and other default parameters. Bioedit Sequence Alignment Editor 7.1.3.0 (Ibis Pharmaceuticals, Inc., Carlsbad, CA, USA) was used for further manual editing. Phylogenetic trees were subsequently constructed by the Maximum likelihood (ML) method using PhyML3.1, based on the best-fit model LG + G estimated by ProtTest2.4. SH-like approximate likelihood ratio (aLRT-SH) supports were used to evaluate the reliability of internal branches. The trees were further edited using the ITOL tool. The identity scores of alignment were extracted using BioEdit software.

### Expression profiles of heat-shock proteins (HSPs) and antioxidant enzymes genes in *A. gifuensis*

Total RNA was extracted using TRIzol reagent (Takara Bio, Tokyo, Japan), as per manufacturer's instructions. The RNA integrity was verified by 1% agarose gel electrophoresis and the quantity was assessed using a Nanodrop ND-2000 spectrophotometer. Then, the cDNA was synthesized from total RNA using PrimeScript™ RT reagent Kit with gDNA Eraser (Perfect Real Time) (Takara, Dalian, China) according to the standard manufacturer's protocol. Gene-specific primers were designed by Primer Premier 5 (PREMIER Biosoft International, Palo Alto, CA, USA), and are shown in Table S1. qPCR was conducted in 20 μl reactions containing 50 × SYBR Premix, Ex Taq (10 μL), primer (10 mM), sample cDNA (0.8 μL), and sterilized ultra-pure grade H_2_O (7.6 μL). Cycling conditions were 95°C for 30 s, 40 cycles of 95°C for 5 s, and 58°C for 30 s. Each sample had three technical replicates and three biological replicates. Relative quantification was performed using the Comparative 2^−ΔΔCT^ method. Transcription levels of these target genes were normalized by 18S RNA, and the normalization of each gene was compared with the expression in female adult at 25°C (Kang et al., 2017a,b).

### Enzyme activity assay

The activities of SOD, CAT, POD, and GST were measured using commercially available assay kits (Nanjing Jiancheng Bioengineering Institute, Jiangsu, China) as described previously (Zhang S. Z. et al., 2015).

### Statistics

The comparison of the performance parameters, gene expression profiles and the activity of antioxidant enzymes between female and male were subjected to Student's *t*-test at *P* < 0.05. A one-way analysis of variance (ANOVA) were used to analyzed different among the different temperature followed by separation of means by the Fisher's protected least significant difference (LSD) test at *P* = 0.05. SPSS 22.0(SPSS Inc., Chicago, IL, USA) was used for data analysis.

## Results

### Performance of *A. gifuensis* exposed to thermal stress

The hatching rate, survival rate, parasitic capacity, longevity and female proportion in offspring of *A. gifuensis* in response to thermal stress were shown in Table 1. The survival rate of female and male were dropped from 100 to 69.67% and 57.67% respectively (Female: *F* = 196.476, *P* < 0.001; Male: *F* = 1,319.976, *P* < 0.001). Consistent with this, the hatching rate of mummified aphid decreased significantly from 100% to 67.67% (*F* = 95.846, *P* < 0.001,). In addition, the survival of female adults in all treatment temperatures were significantly higher than that of male adults at 35°C (*t* = −5.096, *df* = 18, *P* < 0.001). After thermal stress, the longevities of surviving female and male adults were significantly decreased (Female: *F* = 32.916, *P* < 0.001; Male: *F* = 16.766, *P* < 0.001). Furthermore, the parasitic capacity of surviving female adults were significantly depressed as temperature increased (*F* = 25.381, *P* < 0.001). And the female proportion of offspring produced by surviving female adults was significantly affected by temperature (*F* = 44.918, *P* < 0.001).

**Table 1:** The hatching rate, survival rate, parasitic capacity, longevity and female proportion in offsprings of *A. gifuensis* in response to thermal stress.

**Treatment**	**Hatching rate**	**Survival rate**		**Parasitic capacity**	**Longevity/day**		**Female proportion in offspring %**
		**Female**	**Male**		**Female**	**Male**	
25°C	98.00 ± 0.89a	100.00 ± 0.00a	100.00 ± 0.00a	244.50 ± 5.75a	14.40 ± 0.48a	11.30 ± 0.47a	78.50a
30°C	82.51 ± 1.56b	84.01 ± 1.39b	81.00 ± 1.32b	217.60 ± 6.51b	10.30 ± 0.52b	9.80 ± 0.55a	73.50b
35°C	67.67 ± 1.99c	69.67 ± 1.26c	57.67 ± 1.99c	192.70 ± 6.10c	8.50 ± 0.58c	7.20 ± 0.49b	58.00c
40°C	0	0	0	–	–	–	–

*Different letters indicate significant difference among the treatment (P < 0.05)*.

### The identification of HSPs and antioxidant enzyme genes in *A. gifuensis*

**Figure 1:**
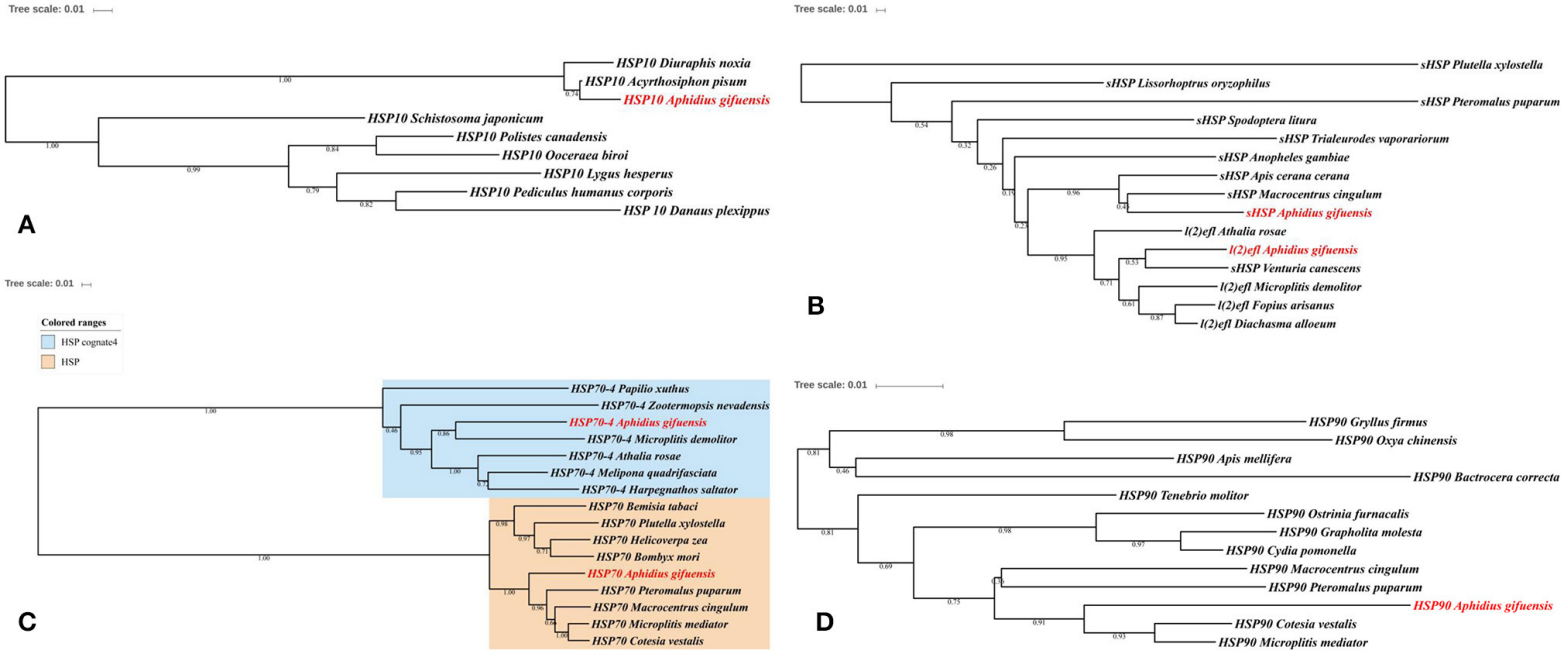
Phylogenetic analysis of HSPs in insects. **(A)** HSP10, **(B)** sHSP, **(C)** HSP70, and **(D)** HSP90.

In this work, we identified six HSPs and 11 antioxidant enzymes, including catalase, superoxide dismutase, peroxidase and glutathione S-transferase (Tables 2, 3). The phylogenetic analysis of HSPs in *A. gifuensis* was shown in Figure 1. All of these genes were clustered into four different HSP groups including sHSP, HSP10, HSP 70s, and HSP90 and presented individually. Especially, *AgifHSP10* were highly conserved with *HSP10* in *A. pisum* (Figure 1A). The identified *AgifsHSPs* in this work showed close relationship with *sHSP* in *Macrocentrus cingulum* and *sHSP* in *Venturia canescens* respectively (Figure 1B). And, HSP70s in this work were classified into two protein families: heat shock proteins (HSPs) and heat shock cognate proteins (HSCs). *AgifHSP70-4* was classified into HSCs, while *AgifHSP70* was clustered into HSPs (Figure 1C). Furthermore, *AgifHSP90* was highly conserved with *HSP90* in *C. vestalis* and *Microplitis mediator* (Figure 1D).

**Table 2:** The identified heat-shock proteins in *A. gifuensis*.

**Gene name**	**Accession number**	**FPKM/M**	**FPKM/F**	**Blast P hit**	***E*-value**	**Identify**
*AgifHSP10*	MG387115	3.22	5.34	NP_001119666.1| heat shock 10kDa protein 1 [*Acyrthosiphon pisum*]	4e-57	99%
*AgifsHSP*	MG387120	1121.40	865.67	AEH05930.1| small heat shock protein [*Apis cerana cerana*]	7e-79	64%
*Agifl(2)efl*	MG387118	16.98	16.51	XP_011308542.1| PREDICTED: protein lethal(2)essential for life-like isoform X2 [*Fopius arisanus*]	9e-45	80%
*AgifHSP*70	MG387117	0.23	0.34	ABV55505.1| heat shock protein 70 [*Microplitis mediator*]	0	89%
*AgifHSP70-4*	MG387116	0.12	0.40	XP_008557093.1| PREDICTED: heat shock 70 kDa protein 4 [*Microplitis demolitor*]	0	74%
*AgifHSP90*	MG387119	4393.18	5658.33	AGF34719.1| heat shock protein 90 [*Cotesia vestalis*]	0	83%

**Table 3:** The identified antioxidant enzyme related genes in *A. gifuensis*.

	**Gene name**	**Accession number**	**FPKM/M**	**FPKM/F**	**Blast P hit**	***E*-value**	**Identify**
Catalase	*AgifCAT1*	MG387104	0.01	0.321	XP_001943641.1 PREDICTED: catalase [*Acyrthosiphon pisum*]	3e-73	99%
	*AgifCAT2*	MG387105	48579.33	36723.67	XP_015120357.1 PREDICTED: catalase [*Diachasma alloeum*]	0	79%
Superoxide dismutase	*AgifSOD1*	MG387106	0.34	0.40	NP_001156243.1 superoxide dismutase [Cu-Zn]-like [*Acyrthosiphon pisum*]	1e-105	100%
	*AgifSOD2*	MG387107	4393.18	5658.33	XP_015110247.1 PREDICTED: superoxide dismutase [Cu-Zn]-like [*Diachasma alloeum*]	7e-80	71%
	*AgifSOD3*	MG387108	3436.74	2929.51	XP_015110252.1 PREDICTED: superoxide dismutase [Cu-Zn]-like [*Diachasma alloeum*]	1e-119	86%
Peroxidase	*AgifPOD*	MG387109	1.12	2.12	XP_012343060.1 PREDICTED: peroxidase [*Apis florea*]	1e-17	55%
Glutathione S-transferase	*AgifGST1*	MG387110	1.20	2.12	XP_006623588.1 PREDICTED: glutathione S-transferase 1-like [*Apis dorsata*]	8e-135	79%
	*AgifGST2*	MG387111	0.71	5.46	NP_001156274.1 glutathione S-transferase [*Acyrthosiphon pisum*]	2e-158	100%
	*AgifGST3*	MG387112	241.49	197.53	AIL29318.1 glutathione S-transferase sigma 1 [*Cnaphalocrocis medinalis*]	5e-107	74%
	*AgifGST4*	MG387113	2.23	4.00	XP_011303377.1 PREDICTED: glutathione S-transferase-like [*Fopius arisanus*]	1e-107	73%
	*AgifGST5*	MG387114	0.15	0.34	NP_001155757.1 glutathione S-transferase omega-1-like [*Acyrthosiphon pisum*]	4e-96	100%

**Figure 2:**
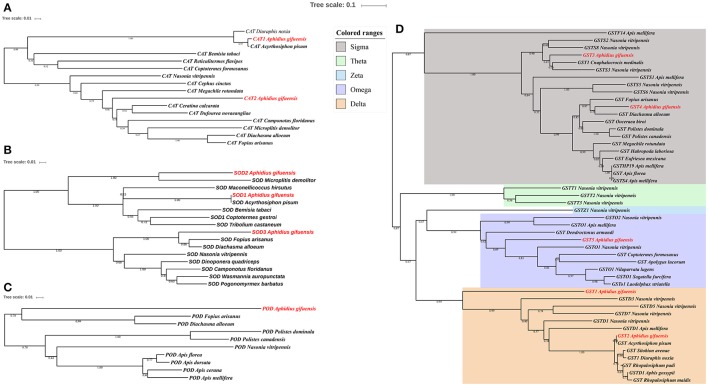
Phylogenetic analysis of antioxidant enzyme genes in insects. **(A)** Superoxide dismutase (SOD), **(B)** peroxidases (POD), **(C)** catalase (CAT), **(D)** glutathione-S-transferases (GST).

The phylogenetic analysis of antioxidant enzyme genes was presented in Figure 2. Interestingly, *AgifCAT1, AgifSOD2*, and *AgifGST2* were clustered with related genes in *A. pisum* respectively. Besides that, *CAT2* showed close relationship with *CAT* in *Ceratina calcarata* (Figure 2A). *AgifSOD2* was highly conserved with *SOD* in *Microplitis demolitor*, while *AgifSOD3* exhibited close relationship with *SODs* in *Fopius arisanus* and *Diachasma alloeum* (Figure 2B). Similar, *Agi*fPOD also showed close relationship with *PODs* in *Fopius arisanus* and *Diachasma alloeum* (Figure 2C). Furthermore, five GST genes were clustered into three different classes: *AgifGST3* and *AgifGST4* belong to Sigma class, *AgifGST5* belongs to Omega class, and *AgifGST1* and *AgifGST2* belong to Delta class (Figure 2D).

### The expression of HSPs in *A. gifuensis* exposed to thermal stress

**Figure 3:**
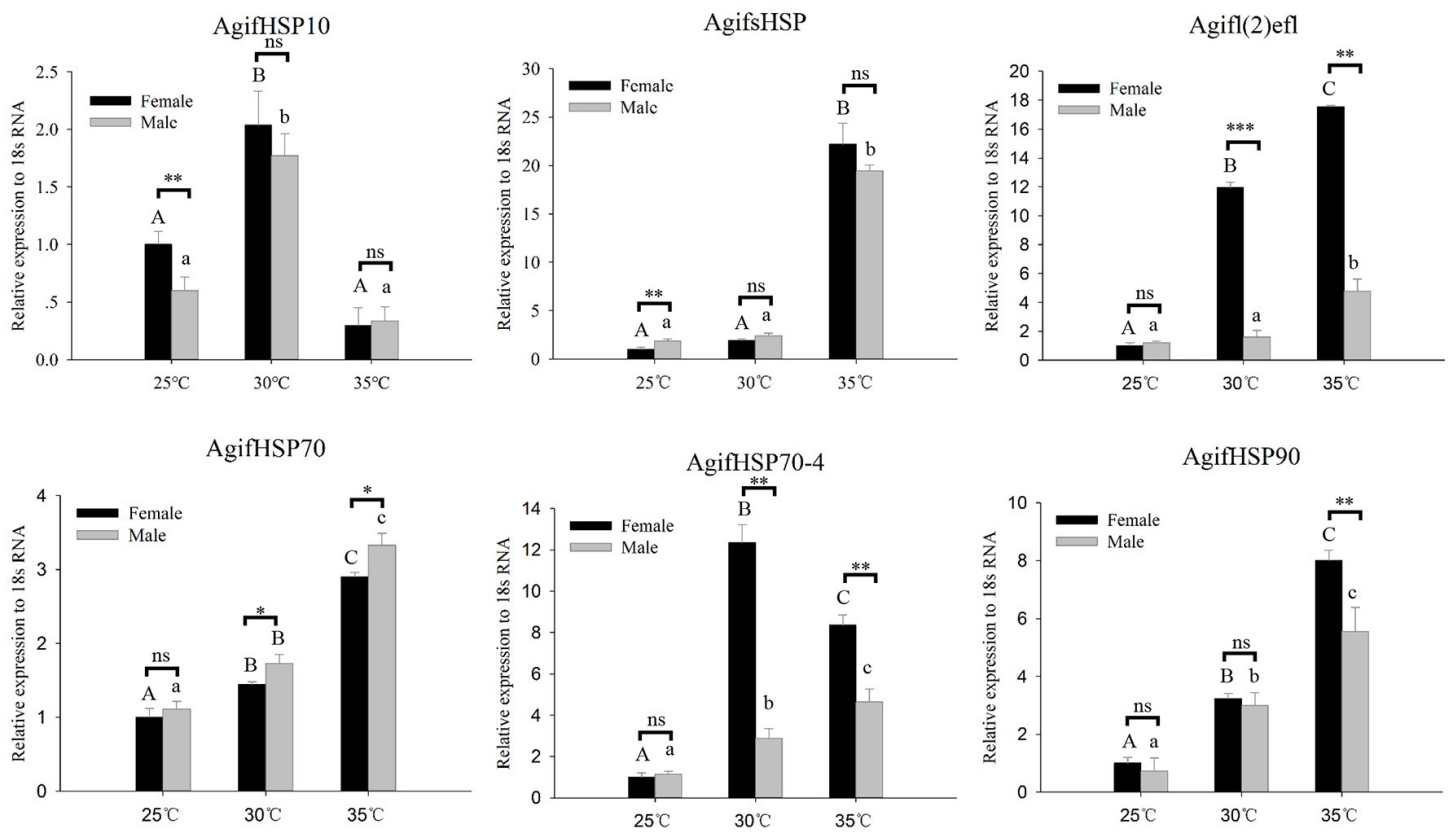
Relative expression of *A. gifuensis* HSPs under the short-term thermal treatment. Different letters over the bars designate a significant difference at *P* < 0.05. And “^*^” means *P* < 0.05, “^**^” means *P* < 0.01, “^***^” means *P* < 0.001.

**Figure 4:**
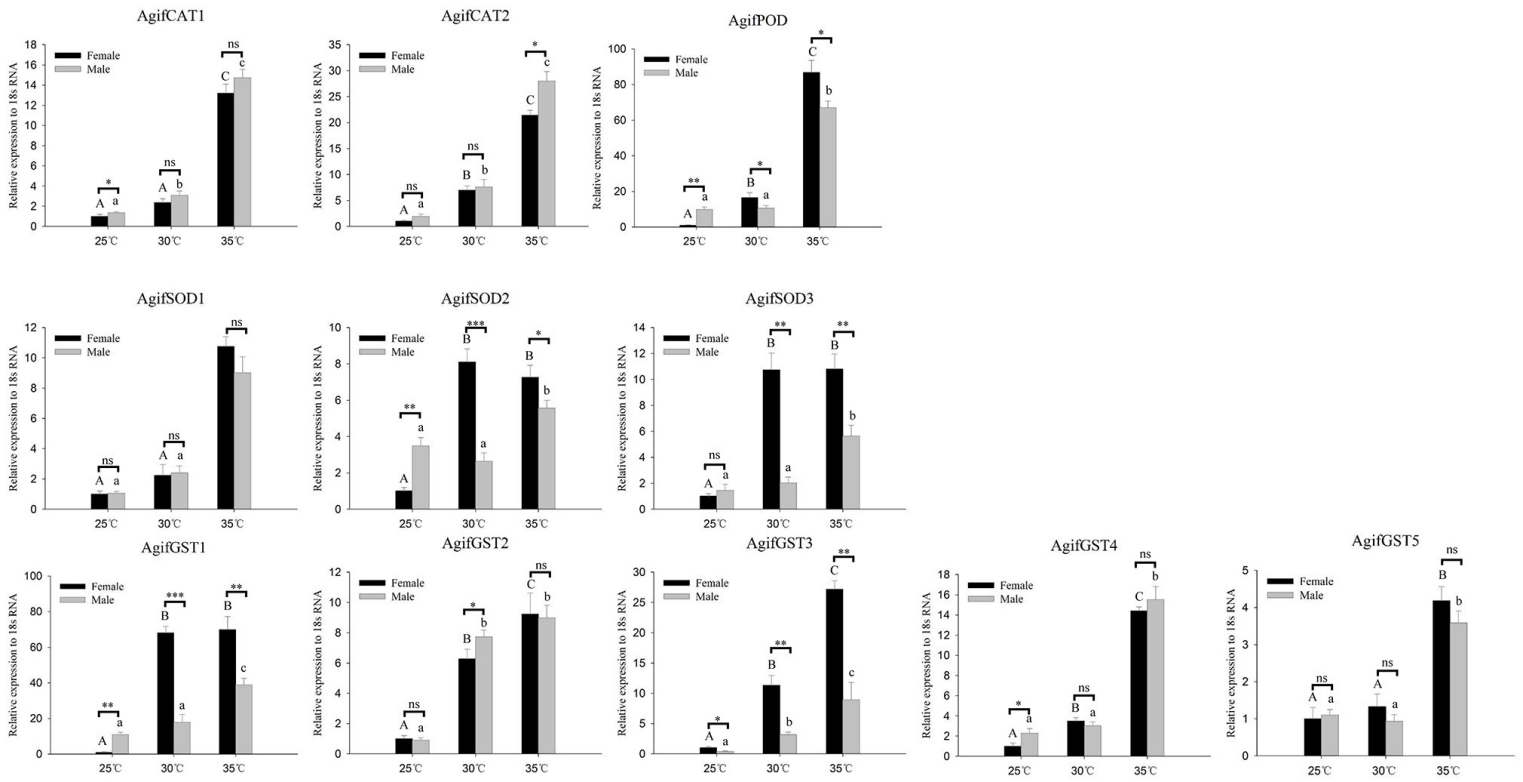
Relative expression of *A. gifuensis* antioxidant enzyme genes under the short-term thermal treatment. Different letters over the bars designate a significant difference at *P* < 0.05. And “^*^” means *P* < 0.05, “^**^” means *P* < 0.01, “^***^” means *P* < 0.001.

To analyze the expression patterns of these HSPs in response to thermal stress, we performed a RT-qPCR experiment to analyze the transcript levels (Figure 3). Under short-term heating, the expression of *AgifHSP10, AgifsHSP, Agifl(2)efl, AgifHSP70, AgifHSP70-4*, and *AgifHSP90* showed an increased trend, whereas *AgifHSP10* first increased and then decreased (Figure 4). At 35°C, the expressions of *Agifl(2)efl, AgifHSP70-4*, and *AgifHSP90* in female were higher than those in male, whereas the expression of *AgifHSP70* at 35°C and the expression of *AgifsHSP* at 25°C exhibited an opposite trend. The expressions of *Agifl(2)efl* and *AgifHSP70-4* in female were also higher than that in male at 30°C.

### The expression of antioxidant enzyme genes in *A. gifuensis* exposed to thermal stress

As for the antioxidant enzymes, we found all of these gene were significantly induced by heat stress whereas there was no significant increase of some genes at 30°C (Figure 4). In both 30° and 35°C exposure treatment, the expression patterns of *AgifPOD, AgifSOD2, AgifSOD3, AgifGST1*, and *AgifGST3* in female were significantly higher than that in male (Figure 4). However, the expression of *AgifPOD, AgifGST1, AgifGST3, AgifGST4, AgifCAT1*, and *AgifSOD2* at 25°C and the expression of *AgifCAT1* at 35°C in female were lower than that in male (Figure 4).

### Antioxidant enzyme activities of *A. gifuensis* in response to thermal stress

**Figure 5:**
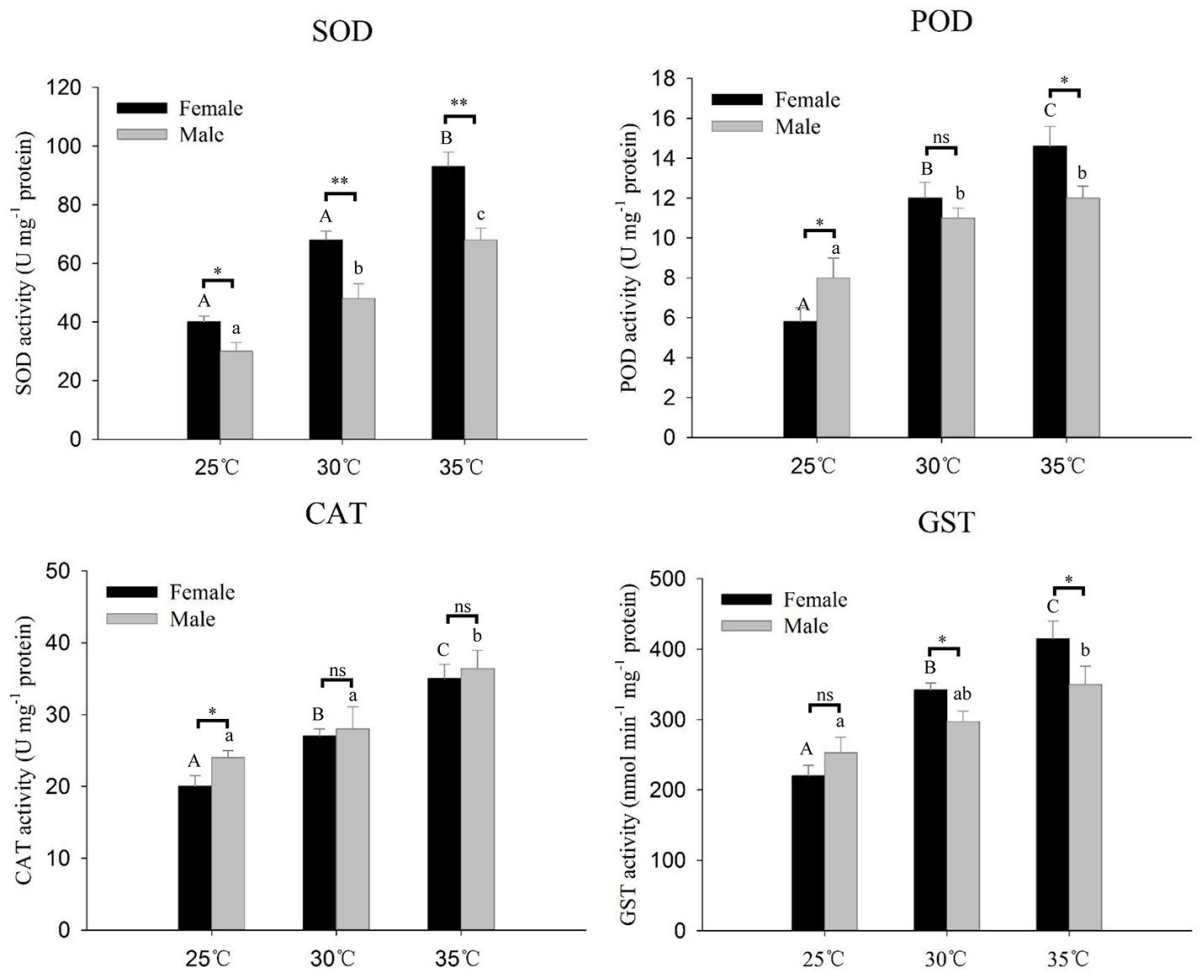
Antioxidative enzyme activities of *A. gifuensis* adults after different levels of heat stress for 1 h. The temperature of 25°C severed as a control. Each value represents the mean (±SE) of five replications. Different letters over the bars designate a significant difference at *P* < 0.05. And “^*^” means *P* < 0.05, “^**^” means *P* < 0.01, “^***^” means *P* < 0.001.

Antioxidant enzyme activities (SOD, CAT, POD, and GST) of *A. gifuensis* in response to thermal stress are presented in Figure 5. All of these four antioxidant enzymes activities were significantly increased along with the increase of temperature (Figure 5). The activities of SOD and GST at 30 and 35°C in female were significantly higher than that in male (Figure 5). However, at 25°C, the activities of CAT and POD in male were significantly higher than that in female (Figure 5).

### Integrated application of aphid natural enemies

**Figure 6:**
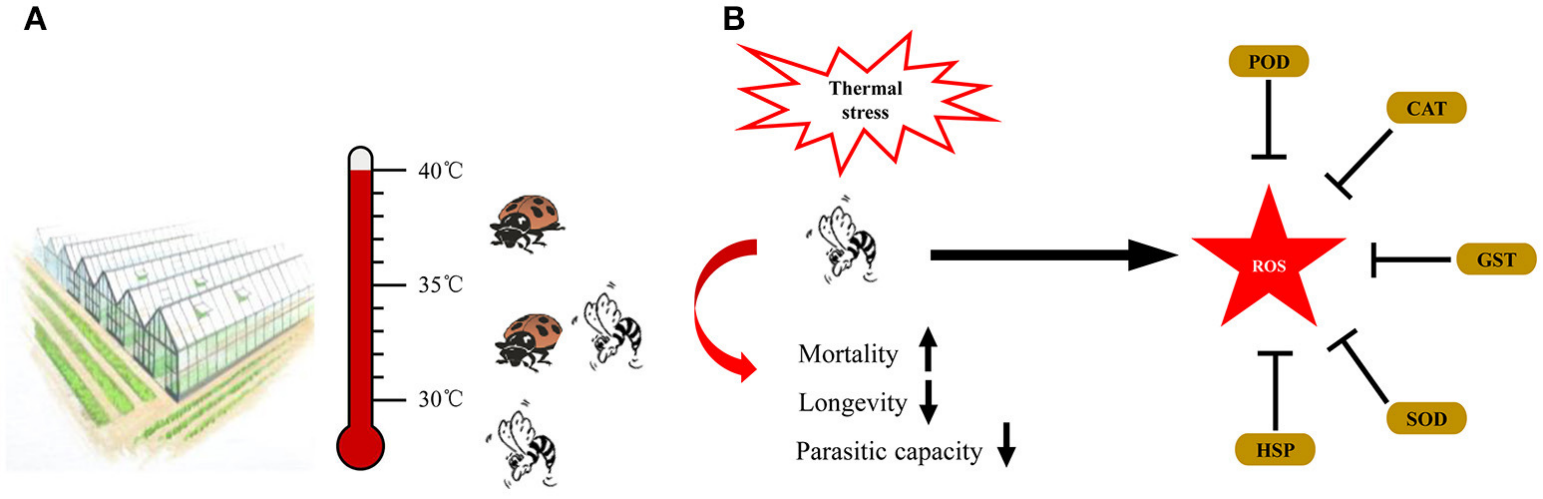
Summary diagram of the integrated application of natural enemies in greenhouse **(A)** and the coordination of HSPs and antioxidant enzyme genes in *Aphidius gifuensis* under thermal stress **(B)**.

Due to the different heat tolerance of *A.gifuensis, A. asychis, A. avenae*, and *P. japonica*, we constructed an integrated application of these natural enemies of aphid (Figure 6A). When temperature under 30°C, we release *A. gifuensis, A. asychis*, and *A. avenae* to control the aphid in greenhouse and filed. When the temperature is from 30° to 35°C, we could use *A. asychis* and *P. japonica* to suppress the quantity of pest aphid. If the temperature above 35°C, *P. japonica* was the best choice for the biological control of aphid.

### The coordination of HSPs and antioxidant enzymes in response to thermal stress

Based on the gene expression and activities of antioxidant enzymes, we hypothesized that under thermal stress, HSPs protect proteins against the denaturation, and antioxidant enzymes scavenge the thermal stress induced ROS to maintain the physiological homeostasis (Figure 6B).

## Discussion

In the application process of natural enemies in eco-agricultural system, thermal stress is one of the key limited factors. In the present work, we not only evaluated the perspective of thermal stress on parasitoid performance but also screened the key potential molecular mechanism involved in this process.

In this work, we found that brief thermal stress negatively affected the parasitoid performance including the hatching rate, survival rate, parasitic capacity, longevity, and female proportion in offspring. Our results showed that female adults were more tolerant to a brief heat stress than male, which was derived from the higher survival rate and longer longevity of female adult in treatment temperatures. The similar results were found in *Aphelinus asychis* and *Aphidius avenae* when they were exposed to a brief heat stress (Roux et al., 2010; Wang et al., 2016). When exposed to 40° and 41.5°C, the mean percent survival of *A. asychis* females was greater than those of males (Wang et al., 2016). However, in *A. asychis*, the longevity of female adults was significantly longer than that of male adults in all treatment temperatures (Wang et al., 2016). Meanwhile, the emergence rate of *A. gifuensis* pupae was strongly affected by the heat stress, which was consistent with the depletion of *Aphidius colemani* and *Trichogramma brassicae* pupae exposed to a brief heat stress aaand (Hoffmann and Hewa-Kapuge, 2000; Colinet and Hance, 2009). In addition, the parasitic capacity of *A. gifuensis* female was negatively affected by the heat stress. This phenomenon was also observed in other parasitic wasps. For example, the number of mummified aphids produced by *A. asychis* females decreased from 413.4 to 71.8 when treatment temperature increased from 25° to 41.5°C (Wang et al., 2016). In this work, our findings highlighted the significance of the negative effects of thermal stress on *A. gifuensis* performances and recommended the suitable application temperature is under 30°C. In addition, there was a highly survival rate from 30° to 41°C in *Propylaea japonica* (Zhang S. Z. et al., 2015). Due to the different heat tolerance of *A.gifuensis, A. asychis, A. avenae* and *P. japonica*, we constructed an integrated application of these natural enemies of aphid. When temperature under 30°C, we release *A. gifuensis, A. asychis* and *A. avenae* to control the aphid in greenhouse and filed. When the temperature is from 30° to 35°C, we could use *A. asychis* and *P. japonica* to suppress the quantity of pest aphid. If the temperature above 35°C, *P. japonica* was the best choice for the biological control of aphid. The integrated application of natural enemies in greenhouse will not only enhance the efficiency of their biological agents but also maximize the benefits through the cost reduction of these biological agents.

Furthermore, we found that at 35°C, *A. gifuensis* female adults performed better than males, which was consistent with the previous results *A. avenae* and *A. asychis*. The potential mechanism of why female exhibited a higher tolerance to thermal stress were: (1) the body size. Numerous studies have revealed that large individuals of both sexes are expected to live longer, to have higher mating success, higher fecundity, produce more daughters, and have a better dispersal ability than small ones (Sagarra et al., 2001; Ellers and Jervis, 2003; Doyon and Boivin, 2005; Santolamazza-Carbone et al., 2007). And, Esperk et al. (2016) have found the body size of *Sepsis punctum* positively affected heat tolerance. And the body size of female adults were larger than male (Wilbert, 1969; De Block and Stoks, 2003; Teder and Tammaru, 2005; Stillwell et al., 2010). Furthermore, female is more sensitive to environment condition (Teder and Tammaru, 2005); (2) the hydrocarbon, wax layer and lipid of insect cuticle (Denlinger and Hallman, 1998). In *Drosophila melanogaster*, the rearing temperature influenced the cuticular hydrocarbons profiles and the cuticular hydrocarbons exhibited the sex-specific profiles (Rajpurohit et al., 2017); (3) response genes. In previous work, several HSPs has been identified to be highly expressed in the ovaries and embryonic tissues of females that is absent in male reproductive structures (Palter et al., 1986; Folk et al., 2006; Krebs and Thompson, 2006; Will et al., 2017). In this work, we analyzed the expression profiles of HSPs and antioxidant enzyme genes to investigate their roles in the tolerance to thermal stress or sex-specific tolerance.

The HSPs are molecular chaperones and comprise a large family of proteins involved in the protection against various forms of cellular stress (King and MacRae, 2015). In this work, we identified five HSPs including *AgifHSP10, AgifsHSP, AgifHSP70, AgifHSP70-4*, and *AgifHSP90* based on the transcriptome sequencing (Kang et al., 2017a). In previous work, the vital roles of sHSPs, HSP70s and HSP90s in responding to thermal and pesticide stresses have been well documented in many insect species (King and MacRae, 2015; Sun et al., 2016). For example, in *Apolygus lucorum*, the expression of *AlucHSC70* was significantly induced by cyhalothrin or extremely high temperature whereas it was decreased significantly in treatments of chlorpyrifos or extreme cold temperature (Sun et al., 2016). Similarly, in *Leptinotarsa decemlineata*, exposure to an extreme temperature of 43°C significantly induced the expression of *LdecHSP70* whereas heat stressed larvae of *L. decemlineata* failed to respond to imidacloprid by producing more HSP70 (Chen et al., 2016). The up-regulation of HSC70 promotes a greater thermal tolerance in *Nilaparvata lugens* (Lu K. et al., 2016). In this work, we found that both *AgifHSP70* and *AgifHSP70-4* were significantly induced under the thermal stress. More interestingly, the expression of *AgifHSP70-4* in female was higher than that in male at 35°C, which was consistent with the higher survival rate of female adult at this temperature. All of these results suggested that *AgifHSP70-4* might be the key factor of temperature resistance in *A. gifuensis*.

Heat-shock protein 90 (HSP90) is a highly conserved molecular chaperone found in all species except for Archaea, which is required not only for stress tolerance but also for normal development (King and MacRae, 2015). For example, *AlucHSP90* was not only an important gene for *A. lucorum* adults in response to extremely high temperature, but also involved in the resistance or tolerance to cyhalothrin, imidacloprid, chlorpyrifos, and emamectin benzoate, especially for female adults to emamectin benzoate and for male adults to cyhalothrin (Sun et al., 2014). In *Acyrthosiphon pisum, ApisHSP83*, which was the homologous genes of HSP90, played pleiotropic roles in embryogenesis, longevity, and fecundity (Will et al., 2017). Knocked down of *ApisHSP83* resulted in the reduction of adult survival and the number of nymphs born per aphid, which appears to be in striking agreement with the role of the homologous HSP90 in the longevity of *D. melanogaster* and *Tribolium castaneum* (Knorr and Vilcinskas, 2011). In *D. melanogaster*, Hsp83 molecular chaperone complex regulated the nuclear import of methoprene-tolerant (Met), which is required for juvenile hormone signal transduction (He et al., 2014). In this work, the expression of *AgifHSP90* was strongly induced by the temperature increasing. And the proportion of female was significantly decreased, which was consistent with *Anisopteromalus calandrae* (Nguyen et al., 2013). All of these results suggested that *AgifHSP90* might be involved in the regulation of longevity and reproduction in *A. gifuensis* under the thermal stress.

Besides HSP70s and HSP90s, sHSP is another well investigated HSPs in insect, which are assumed to play an important role in the heat stress, metamorphosis, normal development, diapause, and immune responses (King and MacRae, 2015). For example, *Csuphsp19.8* and *Csuphsp21.7b* were both up-regulated dramatically by heat and cold whereas *Csuphsp21.5* only be induced by cold stress in *Chilo suppressalis* (Lu et al., 2014). In *Chironomus riparius*, small heat shock protein, *HSP27* was significantly activated by heat stress and xenobiotic exposures including bisphenol A and CdCl_2_ (Martínez-Paz et al., 2014). Additionally, in *D. melanogaster, DmelHSP22* up-regulated not only in oxidative stress condition but also during aging (Morrow et al., 2016). In this work, *AgifsHSP* and *Agifsl(2)efl* were up-regulated in both sexes under a short heat stress, and the higher expression of *Agifsl(2)efl* in female was consistent with the higher resistance of female. All of these results suggested that sHSPs might play an important role in environmental stress response not only thermal stress but also xenobiotic exposures. However, the detail functional investigation is still lacking.

Compared to sHSP, HSP70s and HSP90s, to our knowledge, HSP10 in insects has not been structurally and functionally studied in detail (Jia et al., 2011). In eukaryotes, HSP10, originally identified as a mitochondrial chaperone, now is also known to be present in other places such as cytosol, cell surface, and extracellular space (Jia et al., 2011). In this work, we found that the expression of *AgifHSP10* increased at 30°C in both male and female whereas it decreased at 35°C. Consistent with this result, in *Sitodiplosis mosellana*, the expression of *SmosHSP70, SmosHSC70*, and *SmosHSP90* firstly increased and then decreased with the treatment temperature increasing (Cheng et al., 2016). There are similar trends of *FoccHSP90, FoccHSC701, FoccHSC702*, and *FoccHSP60* in *Frankliniella occidentalis* and *McinHSP70-3* and *McinHSP70-4* in *Melitaea cinxia* (Luo et al., 2015; Lu M. X. et al., 2016). This phenomenon implied that exposed at 35°C destructed the *A. gifuensis* immune system to fail to produce *AgifHSP10*.

Beside the protection of HSPs, organisms are equipped with a comprehensive antioxidant defense system to relieve oxidative stress and remedy the damaged macromolecules produced by the exposure to xenobiotics or thermal stress. SOD is the most important antioxidant enzyme against ROS. In *Panonychus citri* and *Propylaea japonica*, the high temperature exposure increased levels of SOD and GST (Yang et al., 2010; Zhang S. Z. et al., 2015). In this work, all of the expression of *AgifSODs* in female significantly increased at 30°C whereas there was a relatively poor activation of *AgifSODs* in male. Furthermore, at 30° and 35°C, the expression of *AgifSOD2, AgifSOD3, AgifPOD*, and *AgifGSTs* in female were higher than that in male. And the activities of SOD, POD, and GST showed similarly trends of this expression patterns. In previous work, POD has been identified to be involved in the response to thermal stress (Zhang S. Z. et al., 2015). And GST was thought to be participated in the inactivation of toxic lipid peroxidation products accumulated due to oxidative damage and xenobiotics treatment (Qin et al., 2013; Feng et al., 2015). The higher expression and activity of GSTs in female suggested that female with a stronger ability of inactivation of toxic lipid peroxidation products in *A. gifuensis*. Combining with the higher resistance of heat stress in female, we synthesized that the higher activities of SOD, POD and GST are the key factors of the sex-specific heat tolerance. Meanwhile, in *Propylaea japonica*, high temperature exposure also activated the activities of CAT (Zhang S. Z. et al., 2015). Similarly, in this work, the enzyme activity of CAT significantly increased with the increase of temperature, whereas there were no significant difference between female and male at 30° and 35°C, which was consistent with expression profiles of *AgifCAT1* and *AgifCAT2*. In *Antheraea mylitta, C. suppressalis* and *Bombyx mori*, CAT activities also presented a positive correlation with the thermal stress (Cui et al., 2011; Nabizadeh and Jagadeesh Kumar, 2011; Jena et al., 2013). All of these results suggested that the antioxidant enzyme systems play an important role in the antioxidant response under high temperature.

In conclusion, the present study not only highlighted the significance of negative effects of thermal stress on *A. gifuensis* performance but also explored the potential mechanism of antioxidant response in *A. gifuensis*. We found that the protection of thermal damage is coordinated by HSPs and antioxidant enzymes. Based on the heat tolerance ability of many aphid natural enemies, we suggested an integrated aphid management in summer. The integrated application of natural enemies in greenhouse will not only enhance the efficiency of their biological agents but also maximize the benefits through the cost reduction of these biological control agents.

## Author contributions

Z-WK, H-GT, and T-XL designed the research; Z-WK, F-HL, and XL performed research; X-LT, F-HL, W-BY, S-ZZ, and H-GT provided assistance; Z-WK, F-HL, and H-GT analyzed data; Z-WK, X-LT, and H-GT wrote the manuscript; S-ZZ and T-XL edited the manuscript; and Z-WK and X-LT revised the manuscript.

### Conflict of interest statement

The authors declare that the research was conducted in the absence of any commercial or financial relationships that could be construed as a potential conflict of interest.
