# Supplementary material for: The Potential Coordination of the Heat-Shock Proteins and Antioxidant Enzyme Genes of Aphidius gifuensis in Response to Thermal Stress
Source: Front Physiol. 2017 Nov 28;8:976. doi: 10.3389/fphys.2017.00976 (PMC5712418; doi:10.3389/fphys.2017.00976)
Supplement: Supplementary file 1 [file Table1.DOCX]

Table S1. Primers used for target genes and reference genes in RT–qPCR.

| Genes | Primers | Size (bp) | Genes | Primers | Size (bp) |
| --- | --- | --- | --- | --- | --- |
| *AgifHSP10* | F: ATGGCTTCAGTTGCAACCAAGTTC | 109 | *AgifSOD1* | F: TTTGAACGGTGAAGATGTCAAGGG | 164 |
|  | R: TTTCTTGCTCCAGGTCCTACAGCTA |  |  | R: GTGGACCAGAGCTCATACAACCATT |  |
| *AgifsHsp* | F: TCTCAAACTGGTGGGAAGATTTGG | 106 | *AgifSOD2* | F: TAGTATTGGCTTTGGCTGCAACAG | 183 |
|  | R: GCCTGGAGCAATTGTGGTGTCA |  |  | R: TGTGGAAACCATGTTTGCCTGG |  |
| *Agifl(2)efl* | F: CAATAGTTCCAGTGATGTTTCGCG | 162 | *AgifSOD3* | F: ATCAGGTATGCCAATTGATGGTCC | 182 |
|  | R: CAGGTCCAGTAAATATTGGCCTCTG |  |  | R: CCATGTAGGGATTGTAATGAGCACC |  |
| *AgifHsp70* | F: TCCATGATGTTGTTCTCGTTGGTG | 111 | *AgifGST1* | F: ATGGCCCACCATCACTTGCTT | 249 |
|  | R: CCTCGTCGGGGTTTATTGAAAGAT |  |  | R: CACGTGTTTTTGGATCCTTGGGAT |  |
| *AgifHSP70-4* | F: TGAAAGCTGCTATGTTGCTGTTGC | 125 | *AgifGST2* | F: ATGGCCCACCATCACTTGCTT | 249 |
|  | R: GGCAGCAACACCAAGAATACGATT |  |  | R: CACGTGTTTTTGGATCCTTGGGAT |  |
| *AgifHSP90* | F: CAAATCACTCACCAATGACTGGGA | 110 | *AgifGST3* | F: CAATGCCATTTGGACAAGTACCAG | 211 |
|  | R: CAAATGGCATACGACGTGGTACAT |  |  | R: GCTTCGGCATTTGTATCATCCTCA |  |
| *AgifCAT1* | F: TGATGATAACTATACCCAGGCAGGC | 157 | *AgifGST4* | F: TCTGGGTGAACCCATTCGATTTCT | 248 |
|  | R: GCCATCGTCAATTTGAGTGAAATGA |  |  | R: GAGCATTTGCAACCCCATCAAT |  |
| *AgifCAT2* | F: AGTGAGAGATCCACGAGGTTTTGC | 249 | *AgifGST5* | F: TCAACATTACCGGTCTTGGTGAACC | 222 |
|  | R: CCATCAGGTATACCACGATCAGCAA |  |  | R: CGTCATCACCACCAGCAACTTTAA |  |
| *AgifPOD* | F: TGGGGTAGTTCATTAGAAGC | 151 | *Agif18sRNA* | F: TGAATTTGTGTGCCACGCTG | 145 |
|  | R: CCATTAAATAAGGATGTCGA |  |  | R: CAATAAAGAGCACCGCGACG |  |
